# Treatment of Post-traumatic Osteonecrosis in the Distal Tibia With Autologous Bone Grafting: A Case Report

**DOI:** 10.7759/cureus.48214

**Published:** 2023-11-03

**Authors:** Norliyana Mazli, Mohd Yazid Bajuri, Nik Alif Nik Abdullah

**Affiliations:** 1 Orthopedic and Traumatology, Universiti Kebangsaan Malaysia, Kuala Lumpur, MYS

**Keywords:** ankle fracture dislocation, osteochondral injury, autologous bone graft, bimalleolar fracture, tibia, osteonecrosis

## Abstract

Osteonecrosis is a disruption of blood supply to the bone which results in bone cell death. Post-traumatic osteonecrosis of distal tibia rarely happens as compared to osteonecrosis which affects other parts of the musculoskeletal system. We report a case of osteonecrosis of distal tibia in an adult male following an open fracture dislocation of the right ankle. Initial surgery of wound debridement with a temporary external fixator was performed for ankle stabilization. The patient underwent internal fixation once the subcutaneous tissue was deemed suitable. A year later, he had worsening ankle pain which affected his daily activities. Magnetic resonance imaging showed osteonecrosis of the distal tibia, osteochondral injury of the medial tibial plafond, and medial talus with lateral ligament complex injuries. Autologous iliac bone grafting was applied to the distal tibia and a cell-free hyaluronic acid-based scaffold (Hyalofast®) was used to address the bone osteonecrosis and osteochondral injury respectively. Visual analog score (VAS), AOFAS hindfoot score, and ankle range of motion improved at three months and significantly increased after six months and one year post-operatively.

## Introduction

Fracture dislocation of ankles are severe injuries of the ankle and usually result from high-energy trauma. Osteonecrosis of the distal tibia is one of the reported complications, especially in cases of open fracture with severe soft tissue injury [1-4]. The majority of cases lead to worsening collapse of the tibial plafond and compromise the joint function. Therefore, identifying the risk factors is essential for the early identification of osteonecrosis followed by appropriate treatment. Patients with osteonecrosis of the distal tibia will have persistent pain and swelling of the ankle after osteosynthesis. Late detection of osteonecrosis of the distal tibia will result in ankle deformity and joint damage, necessitating either ankle fusion or ankle replacement surgery [2].

The pathogenesis of osteonecrosis is still not clear, potentially stemming from damage to the vulnerable blood supply in the distal tibia following high energy injury, coupled with severe soft tissue damage in cases of open fractures [5]. In atraumatic osteonecrosis, the femoral head, knee, and shoulder are more commonly affected than the distal tibia. The commonest cause of atraumatic osteonecrosis is chronic corticosteroid use, followed by alcohol misuse and smoking [6]. Various methods have been described as the treatment of ankle osteonecrosis, either joint preserving procedures such as core decompression and bone grafting (vascularized and non-vascularized) or joint sacrificing procedures such as talectomy, arthrodesis, and ankle replacement [6,7]. The report highlights a case of osteonecrosis of the distal tibia following open fracture dislocation of the ankle, treated with bone curettage and autologous bone grafting. This article was previously presented as a poster at the 2022 EFAS Congress on October 27, 2022.

## Case presentation

**Figure 1:**
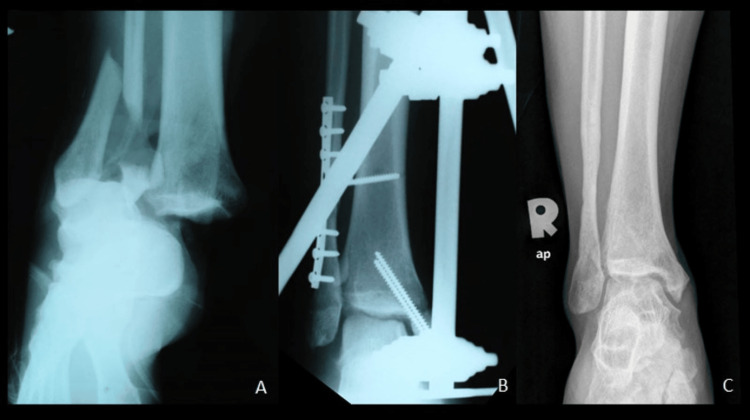
Radiograph during injury showing right ankle bimalleolar fracture with tibiotalar joint dislocation (A), post internal fixation with temporary cross ankle external fixator (B). Radiograph at one year of trauma showed union of bimalleolar fracture with osteochondral injury at anteromedial talar dome (C).

This is a 36-year-old male who was involved in a road traffic accident two years ago and sustained an open bimalleolar fracture right ankle with tibiotalar joint dislocation. He underwent wound debridement and a cross-ankle external fixator for temporary stabilization. Once the condition of the soft tissue was allowed, he underwent another surgery for plating of the right fibula, screw fixation of the right medial malleolus and syndesmosis joint (Figures 1A-1C). After six weeks, the external fixator and syndesmotic screw were removed, and the patient was allowed on partial weight bearing.

He was referred to our center one-year post-trauma with complaints of persistent pain and swelling of his right ankle while walking and standing. The pain was impeding his daily activities, and he was unable to resume working. Upon assessment, he was ambulating with an antalgic gait over the right lower limb. The right ankle was swollen and tender at the anteromedial joint line and lateral ankle. The range of motion was limited, from 0 to 45 degrees of plantarflexion. There was laxity on the anterior drawer test with the ankle in the plantarflexion position.

**Figure 2:**
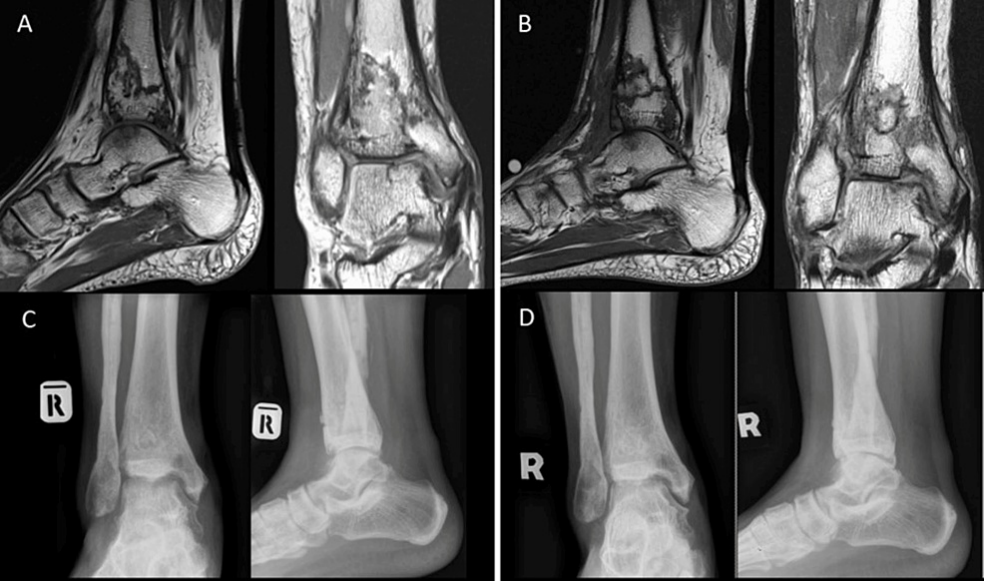
T1-weighted sagittal and coronal view (A). MRI image at one-year post-injury showed abnormal serpiginous signal intensity in the distal tibial metaphysis consistent with bony infarction. Repeated MRI study after six months post bone grafting revealed some area of bone healing (B). Radiograph of the ankle at six months post-surgery (D) showing healing of the bone window as compared to immediate postoperative radiograph (C).

The fibula plate was removed, and he was scheduled for an MRI of his right ankle. The radiograph of the right ankle (Figure 1C) showed a union of the bimalleolar fracture and osteochondral injury over the anteromedial talar dome. MRI of the right ankle (Figure 2A) reported abnormal serpiginous signal intensity in the distal tibial metaphysis consistent with bony infarction, stage IV osteochondral injuries involving the medial talus (anterior and central compartments) and medial tibial plafond measures approximately 1.4 x 1.1cm (AP x W) and 1.7 x 0.9cm (AP x W), respectively. In addition, there was a partial tear of the anterior talofibular ligament (ATFL), deep deltoid ligament, chronic injury to the superficial deltoid ligament, and sprain of the calcaneofibular ligament. The C-reactive protein was 0.22 mg/dL, total white cell count was 6.6 × 10^9^/L.

He underwent operation right ankle debridement, cell-free hyaluronic acid-based scaffold (Hyalofast®) insertion, reconstruction of lateral ankle ligament complex with modified Brostrum-Gould technique, iliac bone grafting of the right distal tibia, percutaneous lengthening of Achilles tendon and platelet-rich plasma (PRP) injection over the medial ankle. The patient was operated on under combined spinal epidural anesthesia and prophylactic antibiotic, intravenous cefuroxime 1.5gram was given. The right lower limb was prepared, and the tourniquet was applied and inflated throughout the procedure. Anterior approach to the right ankle was made and the tissue was dissected in layers to expose the ankle joint. Intraoperatively, there was grade 4 osteochondral injuries measuring 10mm x 10mm over the medial talus (anterior and central compartments) with a subchondral cyst, and over the medial tibial plafond measuring 10mm x 10mm. The cartilage was debrided, microfracture was done and a cell-free hyaluronic acid-based scaffold (Hyalofast®) was inserted with tissue gel to adhere the scaffold to the base.

**Figure 3:**
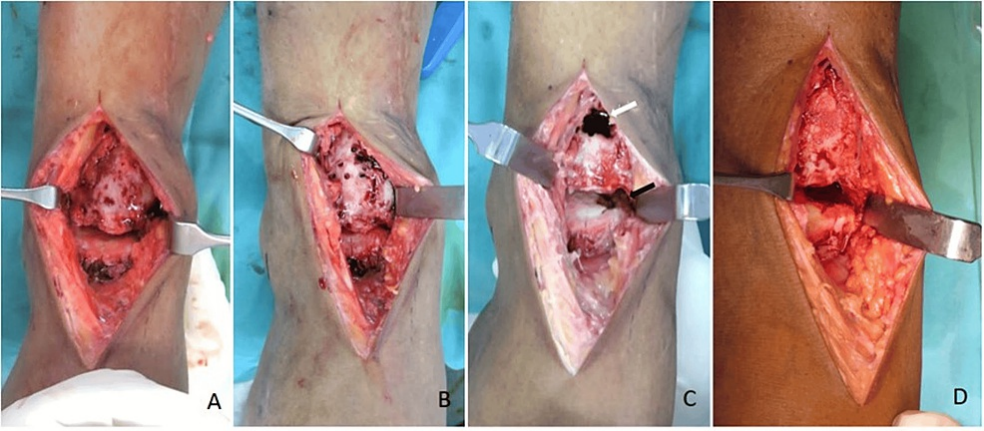
The right ankle joint and distal tibia were exposed (A). Bone window was made with multiple drill holes (B). (C) Chondral defect of the medial talar dome after debridement (black arrow). Necrotic bone curettage and autologous cancellous iliac bone graft were inserted through the bone window (white arrow). The bone window was closed, and the demineralized bone matrix was applied (D).

The area of tibia bone infarction was identified using image intensifiers and the bone window was made at 2 cm above the tibial plafond. The necrotic bone was curetted and washed with normal saline. The medullary cavity was filled with autologous cancellous bone grafts from the iliac crest. The bone window was closed, and the demineralized bone matrix was applied to assist in bone healing (Figures 3A-3D). The ankle joint was closed in layers and dressing was applied. The histopathological examination of the necrotic bone was reported as benign bone tissue with fibrosis, consistent with scar formation. The intraoperative cultures were negative for bacteria and Mycobacterium.

Postoperatively, he was taught non-weight-bearing ambulation with crutches by a physiotherapist. He was discharged on day 3 post-surgery. The wound healed in two weeks without complication. He was allowed partial weight bearing at six weeks post-surgery and full weight bearing after three months. MRI study at six months post bone grafting revealed an area of bone healing at the distal tibia (Figure 2B). Repeated radiographs showed healing of the bone window made intraoperatively (Figure 2D).

During follow-up at six months postoperative, the patient showed significant improvement in symptoms and function. The visual analog score (VAS) improved from 8 to 3, and the Ankle American Orthopaedic Foot and Ankle Society (AOFAS) hindfoot score increased to 78% as compared to 37% before the operation. At one year postoperative, the VAS further reduced to 1 and the AOFAS score increased to 85%. He was satisfied with the overall outcome of the surgery.

## Discussion

Post-traumatic osteonecrosis of distal tibia is a rare complication of fracture dislocation of the ankle. The osteonecrosis may lead to progressive collapse of the distal tibia and loss of ankle joint function [4]. The etiology of distal tibia osteonecrosis in dislocated ankle fractures remains unclear. It is most likely a result of a primary vascular insult or a subchondral fracture following trauma in combination with intraosseous compartment syndrome [5]. The distal tibia is at risk for osteonecrosis due to vulnerable blood supply with limited soft tissue at the ankle joint area. In this case, severe injury to the medial ankle with joint dislocation might injured the extraosseous blood supply to the medial malleolus and distal tibial metaphysis.

Blanke et al. evaluate 28 cases of open dislocated ankle fractures in association with the incident of post-traumatic osteonecrosis [1]. The morphologic characteristics will help to identify the patients with higher risk of developing post-traumatic osteonecrosis following fracture dislocation of the ankle. The study concluded that patients with high energy trauma, type C fibular fracture, complete dislocated talus and grade III soft tissue damage according to Gustillo are recognized risk factors. The authors proposed performing MRI or bone biopsy in patients with those risks followed by surgical decompression. Other factors such as delayed joint reduction, bimalleolar fracture and infection were minor risks.

While the other literature reported cases of osteonecrosis of the distal tibia following Weber C fracture dislocations [1-4], Cinats et al. identified the association of bone necrosis with pilon fractures [8]. They retrospectively evaluate 71 type AO/OTA 43-C pilon fractures and noted that 18 patients developed osteonecrosis at a mean 7.3 months after injury. The study concluded that the fracture patterns are associated with osteonecrosis of the distal tibia, and not related to patient factors such as open injury, smoking, or diabetes [8]. Chakravarty et al. reported post-traumatic osteonecrosis in a patient with high fibular fracture dislocation of the left ankle [3]. The patient was treated conservatively with good recovery.

There are multiple surgical options for addressing distal tibia osteonecrosis which include percutaneous drilling, debridement with vascularized and nonvascularized bone grafting, total ankle arthroplasty and arthrodesis [2,4,9]. Evaluation of a series of nine patients by Assal et al. confirm that open Weber C fractures with ankle dislocation results in post-traumatic osteonecrosis of the lateral tibial plafond [2]. The patients developed complications of valgus ankle deformity owing to further collapse of the lateral tibial plafond which needed surgical reconstruction surgery. Lateral tibial plafond is less vascularized and relatively more sensitive to developed osteonecrosis compared to the medial side. The patients in this series were treated with structural bone grafting with ankle fusion or ankle replacement.

Rajagopalan et al. reported a case of Weber C ankle fracture subluxation which developed osteonecrosis of posterolateral distal tibia at four and a half months after trauma. Percutaneous drilling of the necrotic bone was performed and repeated MRI after four months showed reduction in the area of necrotic bone with no signs of periarticular collapse [4]. During percutaneous drilling of distal tibial osteonecrosis, it is crucial to guide the drill from vascularized bone into the avascular bone to stimulate angiogenesis through the newly created channels [9].

The usage of autologous bone grafting with core decompression for the treatment of post-traumatic osteonecrosis of the femoral head was described in a number of literature with good clinical results [10,11]. The osteonecrosis of distal tibia in this case was treated with bone curettage and autologous cancellous iliac bone grafting. There was significant clinical improvement at six months and one year post surgery, and the patient was satisfied with the outcome. The patient also had stage IV osteochondral injury of the medial talus and medial tibial plafond. The chondral injury was debrided and treated with microfracture and application of cell-free hyaluronic acid-based scaffold (Hyalofast®) to stimulate new cartilage growth. A study of seven patients with osteochondral lesion grade III and IV of the tali that were treated with the same method showed significant improvement of physical function at one year post surgery [12].

## Conclusions

In conclusion, osteonecrosis of the distal tibia may develop following fracture dislocation of the ankle. Early detection by identifying the risk factors can aid in preventing further collapse of the tibial plafond and preserve normal joint function. Autologous bone grafting as treatment of distal tibia osteonecrosis showed promising results with good clinical outcomes.

## References

[1] Blanke F, Loew S, Ferrat P, Valderrabano V, Ochsner PE, Majewski M (2014). Osteonecrosis of distal tibia in open dislocation fractures of the ankle. Injury.

[2] Assal M, Sangeorzan BJ, Hansen ST (2007). Post-traumatic osteonecrosis of the lateral tibial plafond. Foot Ankle Surg.

[3] Chakravarty D, Khanna A, Kumar A (2007). Post-traumatic osteonecrosis of distal tibia. Injury Extra.

[4] Rajagopalan S, Lloyd J, Upadhyay V, Sangar A, Taylor HP (2011). Osteonecrosis of the distal tibia after a pronation external rotation ankle fracture: literature review and management. J Foot Ankle Surg.

[5] Hungerford DS (1981). Pathogenetic considerations in ischemic necrosis of bone. Can J Surg.

[6] Issa K, Naziri Q, Kapadia BH, Lamm BM, Jones LC, Mont MA (2014). Clinical characteristics of early-stage osteonecrosis of the ankle and treatment outcomes. J Bone Joint Surg Am.

[7] Marulanda GA, McGrath MS, Ulrich SD, Seyler TM, Delanois RE, Mont MA (2010). Percutaneous drilling for the treatment of atraumatic osteonecrosis of the ankle. J Foot Ankle Surg.

[8] Cinats DJ, Stone T, Viskontas D, Apostle K (2020). Osteonecrosis of the distal tibia after pilon fractures. Foot Ankle Surg.

[9] Heinen AK, Harris TG (2019). Avascular necrosis of the tibial plafond following rotational ankle fractures. Foot Ankle Clin.

[10] Sharma D, Jhaveri DM, Patel DU, Jha A, Shah P, Golwala P (2019). Pain relief with core decompression and autologous bone graft in osteonecrosis of femoral head in grade 2. Int J Orthop.

[11] Xian H, Luo D, Wang L, Cheng W, Zhai W, Lian K, Lin D (2020). Platelet-rich plasma-incorporated autologous granular bone grafts improve outcomes of post-traumatic osteonecrosis of the femoral head. J Arthroplasty.

[12] Bajuri MY, Sabri S, Mazli N, Sarifulnizam FA, Apandi MH (2021). Osteochondral injury of the talus treated with cell-free hyaluronic acid-based scaffold (Hyalofast®) - a reliable solution. Cureus.

